# Genome-wide transcriptomic analysis identifies candidate genes involved in jasmonic acid-mediated salt tolerance of alfalfa

**DOI:** 10.7717/peerj.15324

**Published:** 2023-05-05

**Authors:** Tianhui Yang, Mei Tian, Ting Gao, Chuan Wang, Xiaochun Wang, Caijin Chen, Weidi Yang

**Affiliations:** 1 Institute of Animal Science, Ningxia Academy of Agriculture and Forestry Sciences, Yinchuan, Ningxia, China; 2 Institute of Horticultural Science, Ningxia Academy of Agriculture and Forestry Sciences, Yinchuan, Ningxia, China; 3 Branch Institute of Guyuan, Ningxia Academy of Agriculture and Forestry Sciences, Guyuan, Ningxia, China

**Keywords:** Salt stress, Transcriptomic analysis, Alternative splicing, Alfalfa, Jasmonic acid

## Abstract

Soil salinity imposes a major threat to plant growth and agricultural productivity. Despite being one of the most common fodder crops in saline locations, alfalfa is vulnerable to salt stress. Jasmonic acid (JA) is a phytohormone that influences plant response to abiotic stimuli such as salt stress. However, key genes and pathways by which JA-mediated salt tolerance of alfalfa are little known. A comprehensive transcriptome analysis was performed to elucidate the underlying molecular mechanisms of JA-mediated salt tolerance. The transcripts regulated by salt (S) compared to control (C) and JA+salt (JS) compared to C were investigated. Venn diagram and expression pattern of DEGs indicated that JS further altered a series of genes expression regulated by salt treatment, implying the roles of JA in priming salt tolerance. Enrichment analysis revealed that DEGs exclusively regulated by JS treatment belonged to primary or secondary metabolism, respiratory electron transport chain, and oxidative stress resistance. Alternatively, splicing (AS) was induced by salt alone or JA combined treatment, with skipped exon (SE) events predominately. DEGs undergo exon skipping involving some enriched items mentioned above and transcription factors. Finally, the gene expressions were validated using quantitative polymerase chain reaction (qPCR), which produced results that agreed with the sequencing results. Taken together, these findings suggest that JA modulates the expression of genes related to energy supply and antioxidant capacity at both the transcriptional and post-transcriptional levels, possibly through the involvement of transcription factors and AS events.

## Introduction

Salinity is one of the most challenging environmental stressors due to its complexity and magnitude (Putnam & Orloff, 2014). Salt stress injures plant photosynthetic systems and limits plant growth and development, even causing plant death (Allu et al., 2014; Mao et al., 2018). Roots are the first and direct organs to perceive soil salinity. Generally, salt stress initially causes osmotic stress of roots, which is a result of physiological drought due to impaired water uptake (Hasegawa, Bressan & Pardo, 2020). These stress responses extend to the whole plant quickly and destroy membrane fluidity and enzyme activity (Arif et al., 2020). Once the salt ions accumulation exceeds the tolerance of plants, plants suffer ion toxicity (Yang & Guo, 2018; Shen et al., 2015). Both osmotic and ion stress lead to the over-accumulation of reactive oxygen species (ROS) (Miller et al., 2010; Van Zelm, Zhang & Testerink, 2020).

Plants initiate genome-wide transcriptional regulation to cope with salt stress. Genes related to osmotic adjustment, ion transport, and antioxidant are up- or down-regulated by salt stress (Gupta & Huang, 2014; Mousavi et al., 2021). For example, plants deploy some low-molecular-weight osmolytes such as proline, to mitigate the adverse effects of salt stress on cell osmoticum (Mansour & Ali, 2017; Yang & Guo, 2018). Previous literature has well proved that the accumulation of proline under salt stress is accompanied by the upregulation of genes encoding its biosynthetic enzymes (Dai et al., 2018). The long-distance upward transport and compartmentalization of Na^+^ need the activation and involvement of genes encoding ion transporters, such as salt overly-sensitive (SOS) genes family, high-affinity K^+^ transporter (HKTs) and Na^+^/H^+^ antiporters (NHXs) (Assaha et al., 2017; Zhu et al., 2016). These genes are regulated to sequester Na^+^ in the vacuole, thereby reducing the cytosolic Na^+^ content and ratio of Na^+^ to K^+^. Furthermore, genes responsible for the scavenging of ROS, including peroxidase, catalase, and superoxide dismutase are activated to resist oxidative stress (Jin et al., 2019; Zhou et al., 2018). In addition, calcium ions (Ca^2+^) are rapidly elevated by salt stress, which is the first response to external salinity (Kiegle et al., 2000). Ca^2+^ sensors and signal transduction components are regulated to transmit and magnify extracellular signals to intracellular (Van Zelm, Zhang & Testerink, 2020). Changes in the transcription of salt-resistant genes are partially dependent on the activation or inactivation of transcription factors (TF). A series of TFs families is evidenced to participate in salt stress responses. For example, overexpression of a NAC transcription factor, OsNAC2, in rice increased salt sensitivity by inducing programmed cell death (Mao et al., 2018). However, another NAC member, NAC45, strengthened rice salt tolerance by enhancing ABA signal transduction (Zhang et al., 2020). These opposite effects of different members among the same TF family indicate the complex regulation through different targets of TF in plant salt response.

Plant hormones have long been thought to be important endogenous molecules that influence plant growth and tolerance or susceptibility to a variety of stressors, including salt stress (Kumar, Rao & Vardhini, 2015). Jasmonic acid (JA) is a phytohormone that influences a series of critical process of plant development and stress responses (Dar et al., 2015, Wang et al., 2020). Salinity stressors have a rapid and substantial effect on JA metabolism and responses by increasing the expression of genes involved in JA synthesis and signaling (Ding et al., 2016, Kuo, Kang & Wang, 2020, Zhao et al., 2020). Furthermore, JA has been proven to neutralize the negative effects of salt stress on plant growth and physiology, by activating the ROS-scavenging system (Qiu et al., 2014), improving photosynthesis (Walia et al., 2007), or simulating the synthesis of osmoregulatory (Anjum et al., 2011).

Alfalfa (*Medicago sativa* L.) is widely cultivated for hay and silage and is well-known as the “king of herbage” for producing a large amount of high-quality pasture. Furthermore, the plant is used as a cover crop and green manure to improve soil health (Klabi et al., 2017). Alfalfa survives well in neutral or mildly saline soils according to its geographical origin in central Asia. However, its yield drops by around 7% for every dS m^−1^ increase in salinity over a particular threshold (Peel et al., 2003). Exogenous application of artificial phytohormone analogs has been reported to improve the salt stress of alfalfa, such as melatonin (Yu et al., 2021). However, the effects of JA on regulating the salt tolerance of alfalfa lack attention. In particular, understanding of JA-regulated genes is limited, restricting a comprehensive assessment of the expression of salt-responsive genes in the majority of forages. We, therefore, performed an RNA-Seq study to compare gene transcription between control, salinity, and salinity plus JA to advance our understanding of phytohormone-regulated gene expression during salt stress.

## Materials and Methods

### Plant materials

Cultivar XinJiangDaYe alfalfa used in this study was provided by professor Zhizhong Cao, Gansu Agricultural University. Alfalfa seeds were sowed in pots with a mixture of peat and vermiculite (v:v, 2:1) and placed in the artificial climate chamber. The growth condition was as follows: 30 °C at day and 25 °C at night, 16 h light and 8 h darkness, a relative humidity at 70%. 3-weeks old plants with identical growth characteristics were transplanted into 1/2 Hoagland nutrient solution and treated with salt (S, 150 mM), Jasmonic acid (J, 5 μM), or salt+JA (JS) after 1-week adaptional growth. The same nutrient solution without salt or JA was used as the control (C). To perform transcriptomic analysis, root samples were collected from three biological replicates after 24 h of treatment.

### Isolation of RNA and transcription in reverse

The total RNA was isolated using the Plant total RNA purification kit (Gmbiolab. Co., Ltd, Dali City, Taiwan). RNA degradation and contamination were assessed using 1% agarose gels. To assess the purity and quantity of RNA, a NanoPhotometer spectrophotometer was used (IMPLEN, Calabasas, CA, USA). The input material for RNA sample preparations was 5 g of RNA per sample. Total RNA (500 ng) was reverse transcribed to cDNA using a ReverTra Ace qPCR RT Kit with genome-DNA-removing enzyme (Toyobo, Osaka, Japan).

### Construction of a cDNA library and sequencing

After separating the total RNA, ribosomal RNA was removed to get messenger RNA (mRNA). The RNA was then fragmented using a fragmentation buffer. cDNA was generated using random hexamers containing dNTPs (dUTP, dATP, dGTP, and dCTP) and DNA polymerase I, and then purified using AMPure XP beads. The purified double-stranded DNA was then subjected to end repair. To create the final cDNA library, a tail well was introduced to connect the sequencing adaptor, AMPure XP beads were used for fragment size selection, and PCR enrichment was performed. The sequence information was uploaded in figshare (DOI 10.6084/m9.figshare.21628505).

### Library quality assessment

After the development of the library, its quality was assessed to confirm that it met sequencing standards. To complete the library inspection, the detection techniques include: (1) the use of Qubit2 for further integrity testing; and (2) the -PCR method to appropriately estimate the effective concentration of the library (library effective concentration >2 M). Following validation of the onboard sequencing library, several libraries were pooled and sequenced on the Illumina HiSeq platform based on the desired off-board data volume. The approach was then proceeded by a bioinformatics analysis in which off-board data was filtered using FASTP to produce Clean Data (Chen et al., 2018). (1) Removal of amplicons with adapters; (2) removal of paired reads when the N content in any sequencing read surpasses 10% of the total number of bases in the read; and (3) removal of paired reads when any sequencing read contained low-quality (Q-20) and the total number of bases in the read exceeded 50% of the total number of bases in the read. Additional structural level analyses were performed on the resulting clean mapped data.

### Alignment of data, genes expression profiles, and differentially expressed genes

The clean reads were aligned to the gene set and compared to the reference genome using HISAT2 software to calculate the mapping event on both the sense and antisense strands. We used the following method for calculating FPKM (Fragments Per Kilobase of Transcript per Million Mapped Fragments) as a measure of transcript or gene expression levels: FPKM = mapped fragments of transcript/Total Count of mapped fragments (Millions) × Length of transcript (kb). To identify differentially expressed genes (DEGs), the raw counts were used as input and analyzed with the DESeq R package (Varet et al., 2016). The thresholds for identifying DEGs were false discovery rate (FDR) <0.05 and |log2Fold Change| ≥ 1. Gene expression comparisons were conducted among different treatments.

### Gene expression cluster analysis

Cluster analysis is used to determine the expression patterns of DEGs under different treatments, and to identify the functions of unknown genes or unknown functions of known genes with the same or similar expression patterns.

Cluster analysis was conducted to investigate gene expression patterns under different treatments. The FPKM values were first centralized and normalized, and then K-means clustering analysis was done. The same class of genes had similar trends under different experimental treatments and may have similar functions.

### Functional annotation and enrichment analysis of differentially expressed genes

The GO function classification was evaluated and visualized, hypergeometry was assessed, and validation and screening of genes enriched with GO significant terms were performed. Finally, DEGs were subjected to the Kyoto Encyclopedia of Genes and Genomes (KEGG) pathway enrichment analysis using the KOBAS online software and statistical analysis. The DEGs were further functionally annotated by comparing the protein or cDNA sequence to the KOG database and then obtaining the annotation from the KOG database.

### Variants of a single nucleotide and alternative splicing

We assessed the expression of alternative splicing events in biologically replicated samples using rMATS, estimated the *P* value using the likelihood-ratio test to indicate the difference between the two sets of samples, and then applied Benjamini Hochberg Methods. FDR was obtained by utilizing multiple hypothesis testing to adjust the *P* value. Differential events were defined as alternative splicing events with an FDR less than 0.05.

### Validation of genes and expression analysis

To confirm the results, selected DEGs were subjected to real-time quantitative PCR (q-PCR) using primers identified by Primer Premier software (Premier Biosoft International, Palo Alto, CA, USA) (Table S1). RT-qPCR was used to amplify cDNAs in a final volume of 20 μL, which included 2 μL of cDNA, 10 μL of SYBR Green master mix with low Rox, 0.5 μL forward and reverse primers, and 7 l nuclease-free water. On an ABI Quantstudio 6 Flex real-time PCR apparatus, 50 °C for 20 s, 95 °C for 10 min, followed by 45 cycles of 3 min at 94 °C, 15 s at 94 °C, 15 s at 58 °C, and 20 s at 72 °C were used to standardize amplification (Applied Biosystems, Foster City, CA, USA). The housekeeping gene, *MsACTIN2* was used as an internal control for normalizing gene expression. To ensure reproducibility and reliability, q-PCR analysis was performed on three independent biological replicates.

### Analytical statistics

A pairwise comparison of phenotypic and relative expression levels between samples was performed using Duncan’s multiple range test with a significance threshold of 0.05. All tests were carried out with IBM SPSS Statistics for Windows 19.0 (IBM Corp, Armonk, NY, USA).

## Results

### Transcript profile of DEGs in the root of alfalfa under different treatment

**Figure 1:**
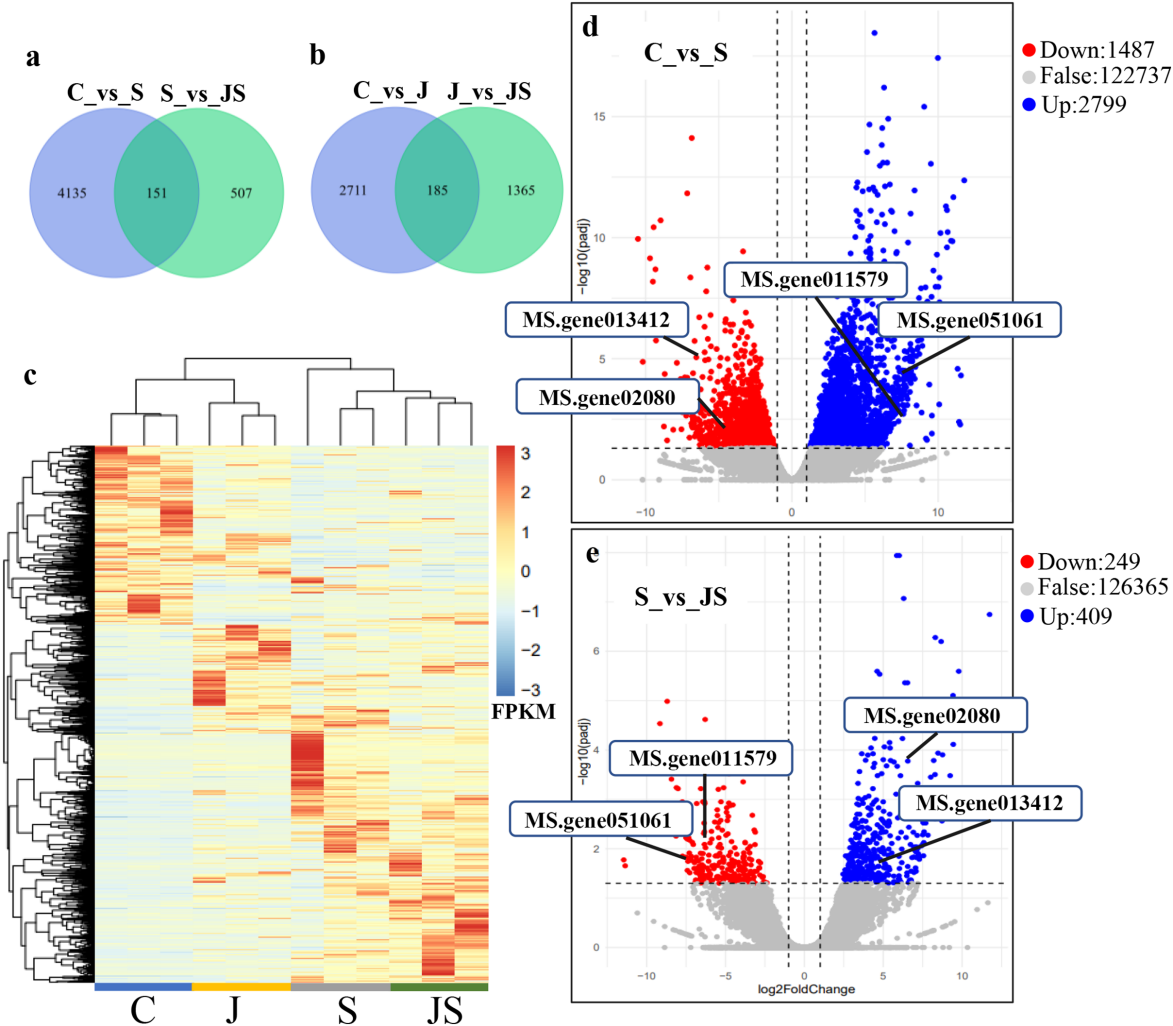
Overview of differentially expressed genes (DEGs). Venn diagrams depicting the overlap of up-regulated genes (A) and down-regulated genes (B) induced by salt and JA+salt treatment. Numbers of DEGs exclusively regulated by certain treatment were shown in one circle. Numbers of DEGs common regulated by salt and JA+salt were shown in overlapping region. (C) A heatmap showing the relative expression level of DEGs under different treatments. (D & E) Volcano Plots showed genes differentially expressed in C_vs_S (D) and S_vs_JS (E) comparisions. C_vs_S: salt (S) treatment compared to control (C). S_vs_JS: JA+salt (JS) treatment compared to salt treatment.

In order to analyze the effects of JA on the salt stress response, the differentially expressed genes (DEGs) were compared between C_vs_S and S_vs_JS, as well as between C_vs_J and J_vs_JS. There were 4,286 and 2,896 genes that were significantly regulated by salt and JA treatment when compared to control, respectively. C_vs_S and S_vs_JS shared 151 genes and C_vs_J and J_vs_JS shared 185 genes. Importantly, 507 and 1,365 genes were detected exclusively in S_vs_JS and J_vs_JS analysis respectively (Figs.1A and 1B). Heatmap of the FPKM of all DEGs obtained by pairwise comparison further revealed that a series of genes were specifically regulated by JA and salt combined treatment (Fig. 1C, Table S2). We focused on the DEGs regulated by salt alone relative to control and JS relative to S. Volcano plots revealed that most DEGs showed in C_vs_S and S_vs_JS comparisions (Figs. 1D and 1E). Remarkably, a set of genes were further altered when JA was added to salt stress compared to salt stress alone (Fig. 1E, Table S2).

**Figure 2:**
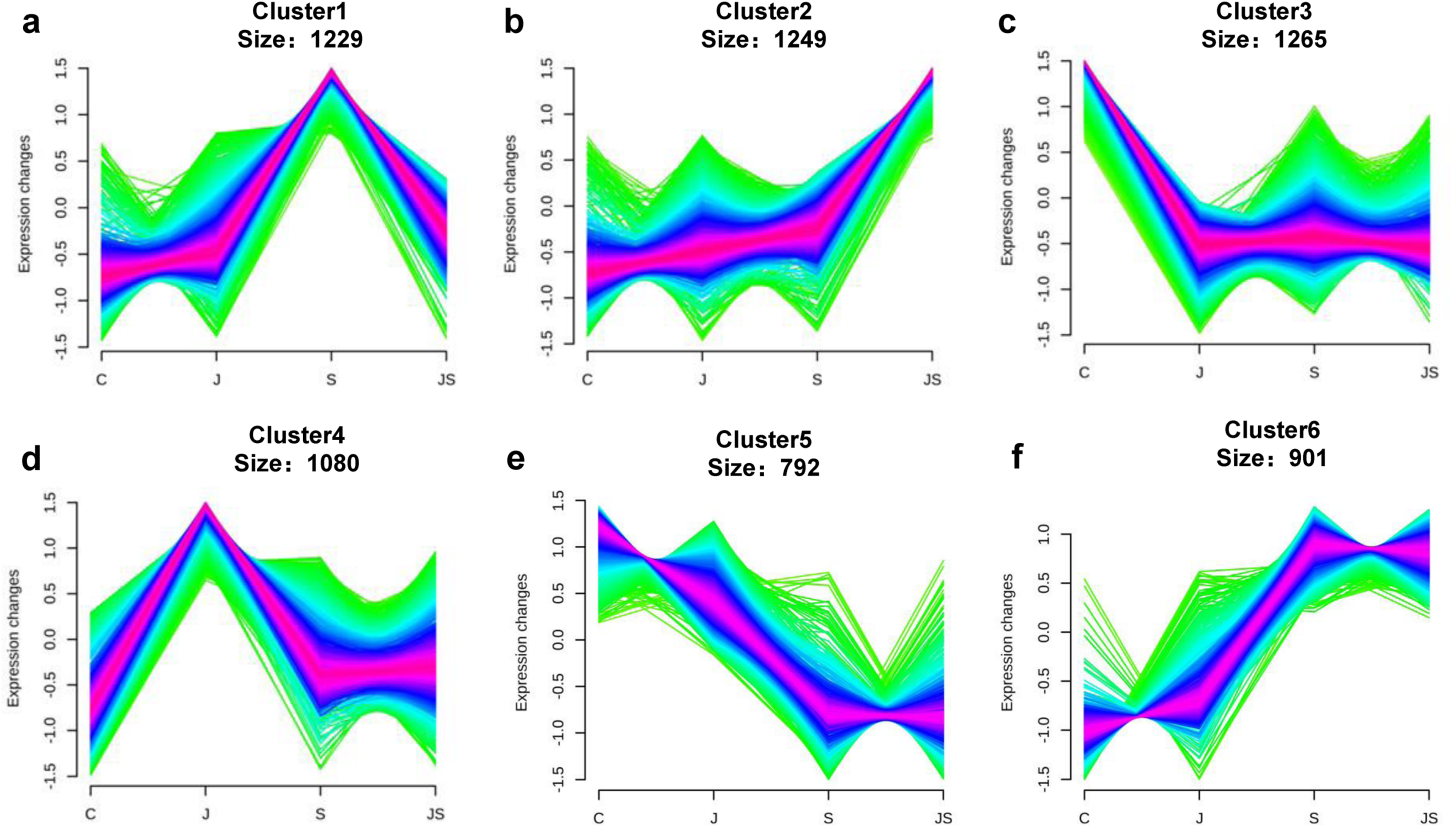
Kmeans cluster analysis of DEGs. DEGs were mainly divided into six groups according to their expression pattern (A–F).

Transcription pattern analysis of all DEGs was analyzed by kmeans and six major expression patterns were identified. Total of 1,129 genes in cluster 1 showed upregulation under salt stress, whereas downregulation under JS conditions compared to S (Fig. 2A). Cluster 2 contains 1,249 genes which were upregulated by salt treatment alone and further induced by JS treatment (Fig. 2B). Expression levels of genes in other four clusters were equal under S and JS conditions (Figs. 2E and 2F). In addition, the DEGs regulated by both S and JS treatment but in opposite directions were analyzed. As shown in Table S3, there were 74 genes were upregulated by salt stress, which were compromised by JS treatment. Some of them were related to plant growth response, such as two *Expansin* genes (MS.gene011579, MS.gene051061, marked in Figs.1D and 1E). Meanwhile, 28 genes down-regulated by salt stress were upregulated in S_vs_JS analysis, such Fe-Mn superoxide dismutase (Fe-Mn SOD, Gene ID: MS.gene013412, marked in Figs.1D and 1E) and peroxidase family protein (POD, Gene ID: MS.gene02080, marked in Figs.1D and 1E). Meanwhile, 42 were commonly upregulated in both C_vs_S and S_vs_JS analysis, such as some salt-responsive TFs (bZIP, MS.gene07054; ERF, MS.gene50632) and an auxin efflux carrier (PIN-LIKES 7, MS.gene86524). Another seven genes were commonly downregulated.

### Functional annotation for DEGs

**Figure 3:**
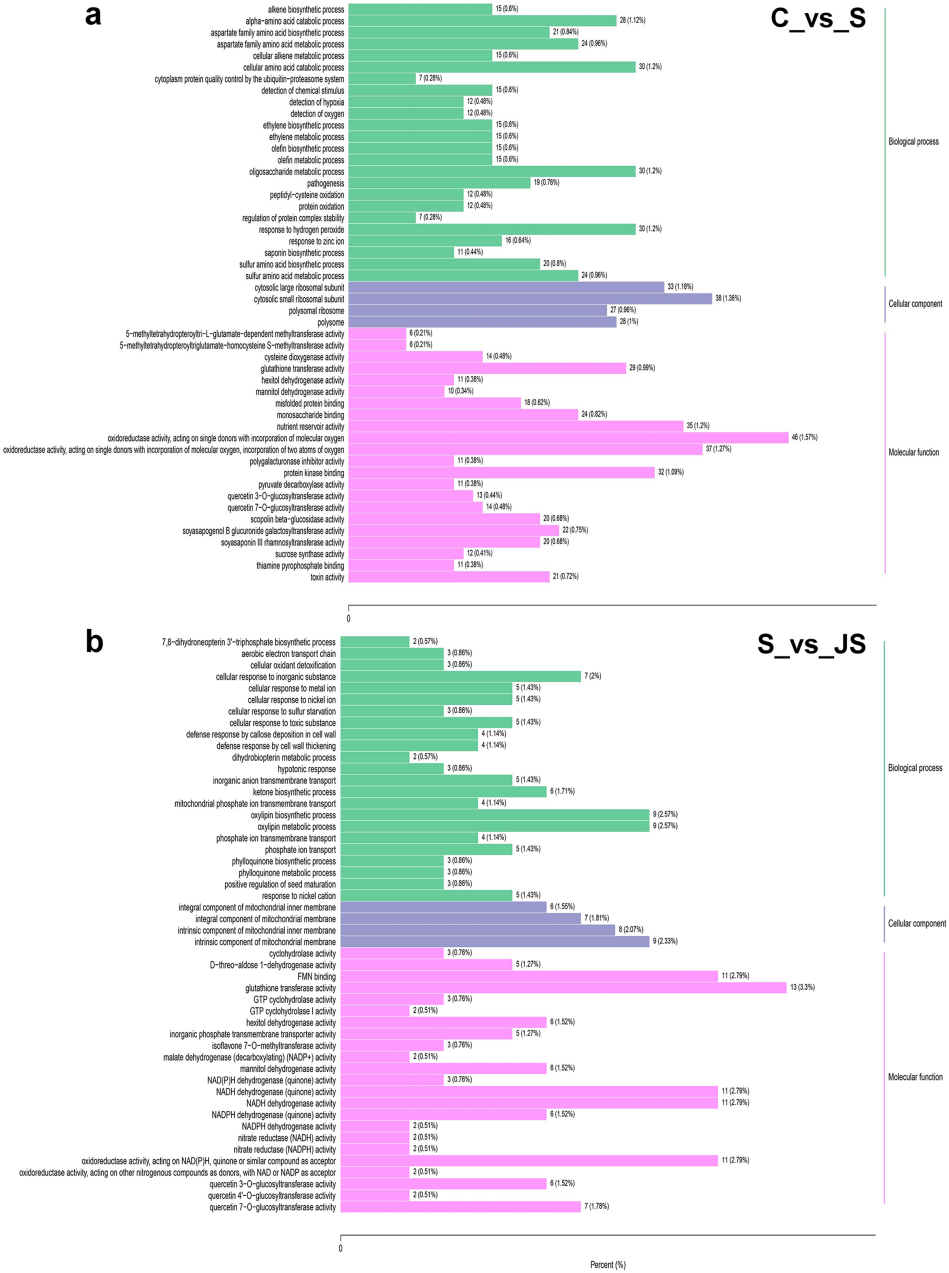
Gene Ontology (GO) enrichment analysis of DEGs in C_vs_S (A) and S_vs_JS (B) groups. C_vs_S: salt (S) treatment compared to control (C). S_vs_JS: JA+salt (JS) treatment compared to salt treatment.

To identify critical gene functions and pathways in large collections of DEGs, we performed a gene set enrichment analysis and retrieved individual genes from overrepresented gene ontology (GO) categories. For each treatment, we looked at the top 50 enriched ontologies. Intriguingly, most ontologies enriched in C_vs_S and S_vs_JS were different, excluding glutathione transferase activity, hexitol dehydrogenase activity, and mannitol dehydrogenase activity in molecular functions (Fig. 3). In detail, the most significant ontology in salt-only regime was a biological process with 28 enrichments. Response to hydrogen peroxide, cellular amino acid catabolic process, and pathogenesis had the most significant number of genes (30), followed by the alpha amino acid catabolic process, which had 28 genes. The molecular function was the second most annotated ontology, with 22 classifications. The two enrichments were oxidoreductase activity, acting on single donors with the incorporation of molecular oxygen and two atoms of oxygen, with 46 and 37 genes, respectively. Nutrient reservoir activity and protein kinase binding were the following enrichments with 35 and 32 genes, respectively (Fig. 3A). In S_vs_JS analysis, biological process and molecular function ontologies showed an equal number of enrichments (23). Remarkably, the enriched biological process in S_vs_JS analysis was completely different from that in C_vs_S analysis. Genes related to oxylipin biosynthetic and metabolic process, and cellular oxidant detoxification were the most significantly enriched by JS treatment. Glutathione transferase activity was the most enriched molecular function (13 genes), followed by FMN binding, NADH dehydrogenase (quinone) activity, NADH dehydrogenase activity, and oxidoreductase activity, acting on NAD(P)H, quinone or similar compound as acceptor (Fig. 3B).

**Figure 4:**
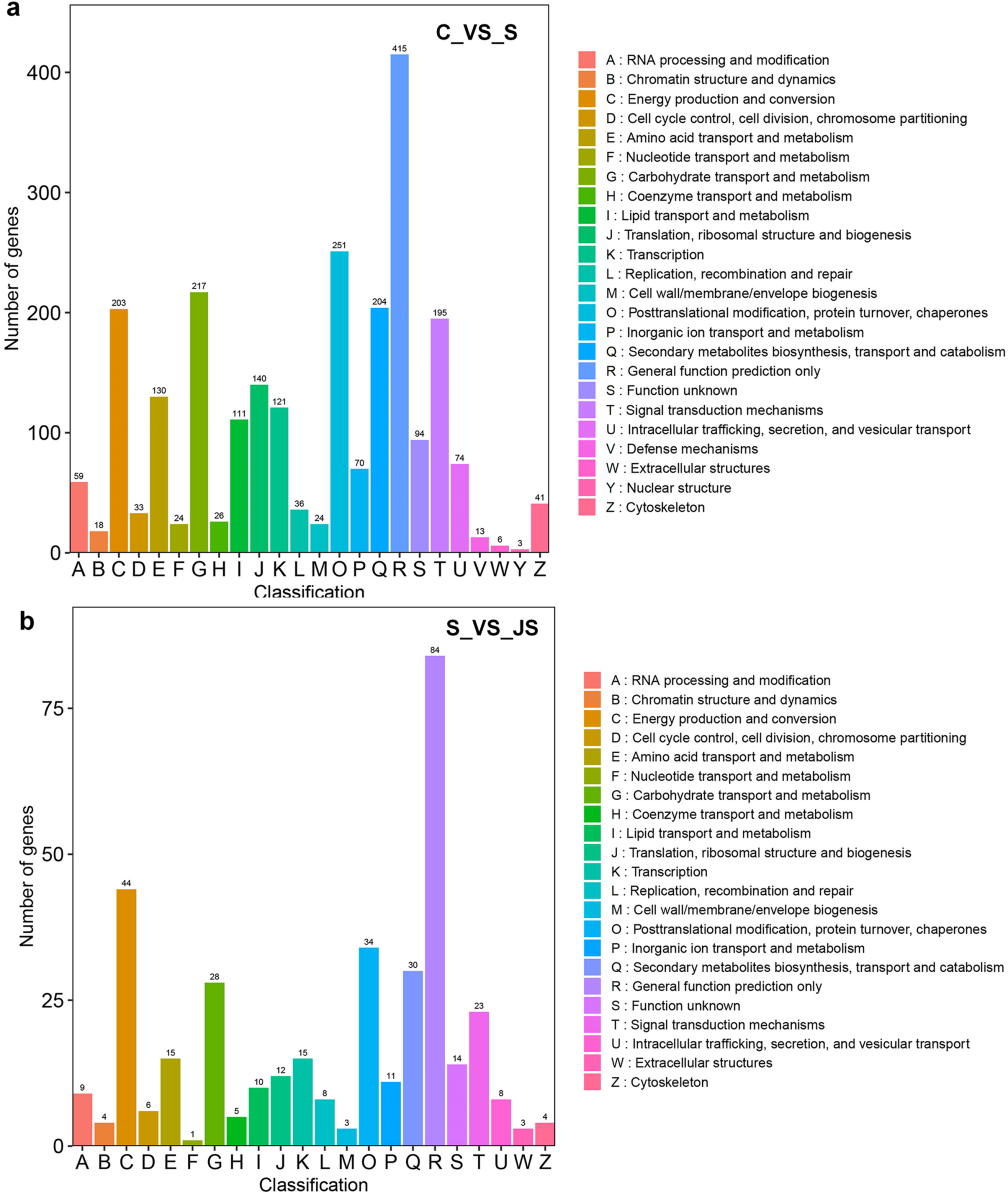
KOG enrichment analysis of DEGs in C_vs_S (A) and S_vs_JS (B) groups. C_vs_S: salt (S) treatment compared to control (C). S_vs_JS: JA+salt (JS) treatment compared to salt treatment.

DEGs from the KOG database were concurrently classified into 26 functional classes. The most common category was general function prediction, with 406 and 84 genes in the C_vs_S and S_vs_JS analysis, respectively (Figs. 4A and 4B). Control of the cell cycle, cell division, chromosomal partitioning, and glucose transport and metabolism were all significantly enriched in C_vs_S and S_vs_JS analysis (Figs. 4A and 4B). A total of 13 genes controlling defense mechanisms, and three genes related to the nuclear structure were only enriched in C_vs_S analysis (Fig. 4A).

**Figure 5:**
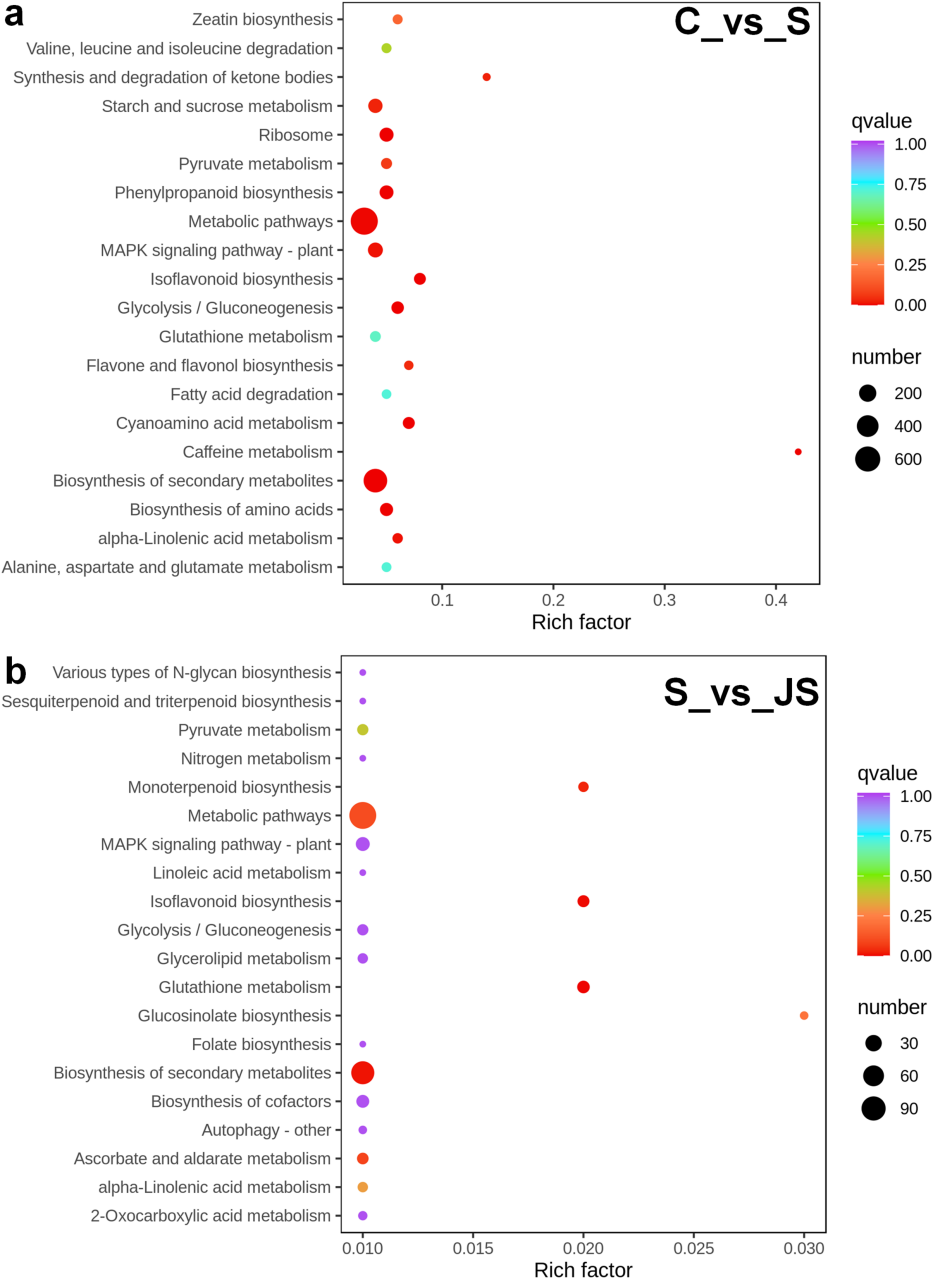
Scatter plot showing Kyoto Encyclopedia of Genes and Genomes (KEGG) enrichment analysis of C_vs_S (A) and S_vs_JS (B) groups. C_vs_S: salt (S) treatment compared to control (C). S_vs_JS: JA+salt (JS) treatment compared to salt treatment.

**Figure 6:**
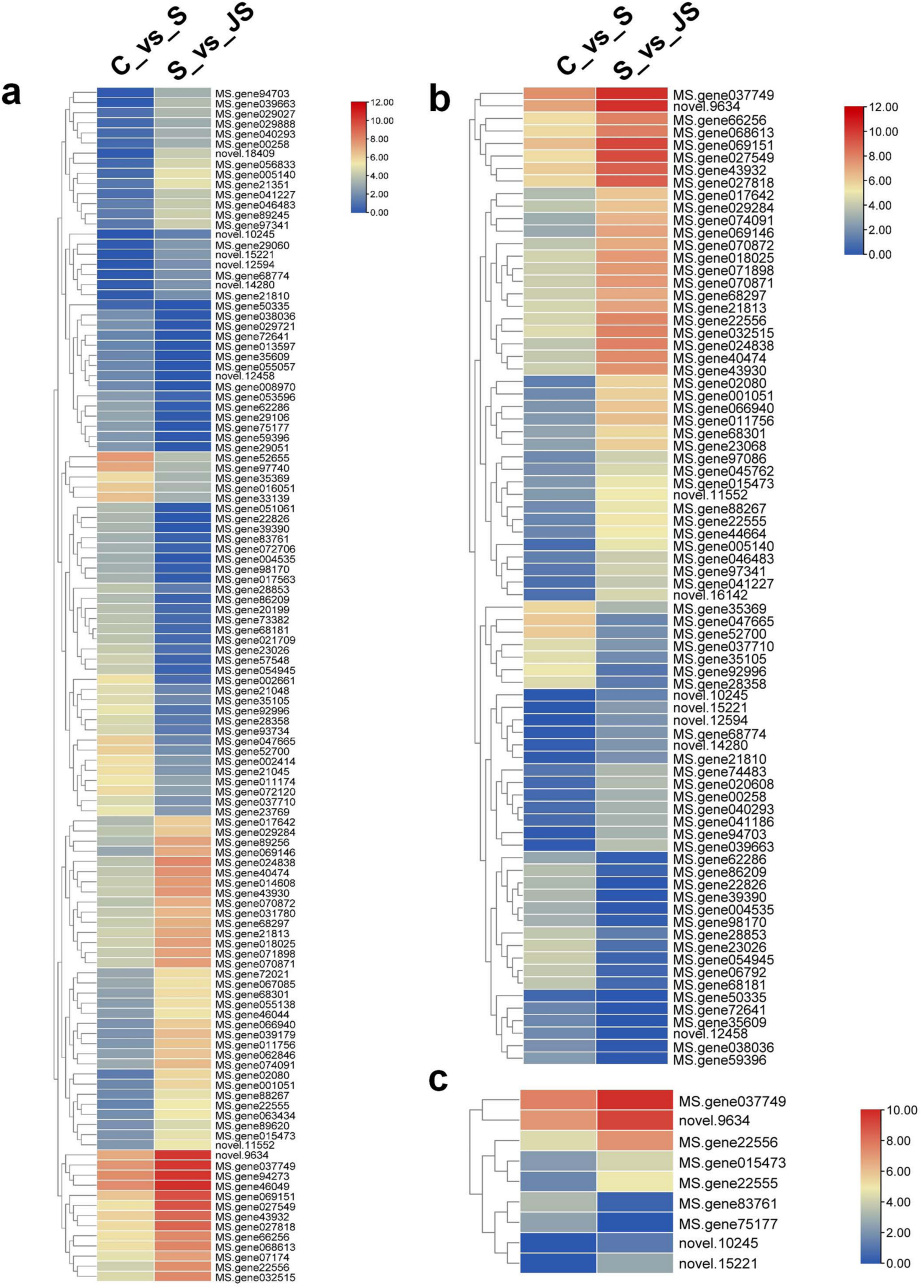
Heatmaps showing relative expression of DEGs enriched in metabolic pathway by KEGG analysis of C_vs_S (A) and S_vs_JS (B) groups. (C) Relative expression of DEGs enriched in ascorbate and aldarate metabolism exclusively in S_vs_JS group.

A KEGG pathway analysis of frequent DEGs was undertaken to better understand their functional relevance in salt response. Results showed that the most significantly enriched pathways were metabolic pathways (737 in C_vs_S and 119 in S_vs_JS) and secondary metabolite biosynthesis (509 in C_vs_S and 79 in S_vs_JS) in both C_vs_S and S_vs_JS comparisons. In addition to the shared enriched-pathways, there were some specific pathways enriched exclusively in C_vs_S or S_vs_JS comparisons. For example, genes involved in starch and sucrose metabolism (106) and ribosome (100) were enriched when comparing S to C, rather than comparing JS to S. Whereas 10 genes related to ascorbate and aldarate metabolism were regulated only by JS treatment (Fig. 5). We then focused on the genes regulated by JS relative to S. DEGs in metabolic pathways and secondary metabolite biosynthesis, the common-enriched pathways, were analyzed in S_vs_JS comparison. As shown in Figs. 6A and 6B, DEGs in the same pathway showed different regulatory patterns, roughly half upregulated and half downregulated. Ascorbate and aldarate are essential antioxidants, and genes related to their metabolism were specifically detected by comparing JS to S. Intriguingly, seven genes in this pathway were upregulated by JA addition to salt treatment (Fig. 6c).

### Discovery of alternative splicing events

**Figure 7:**
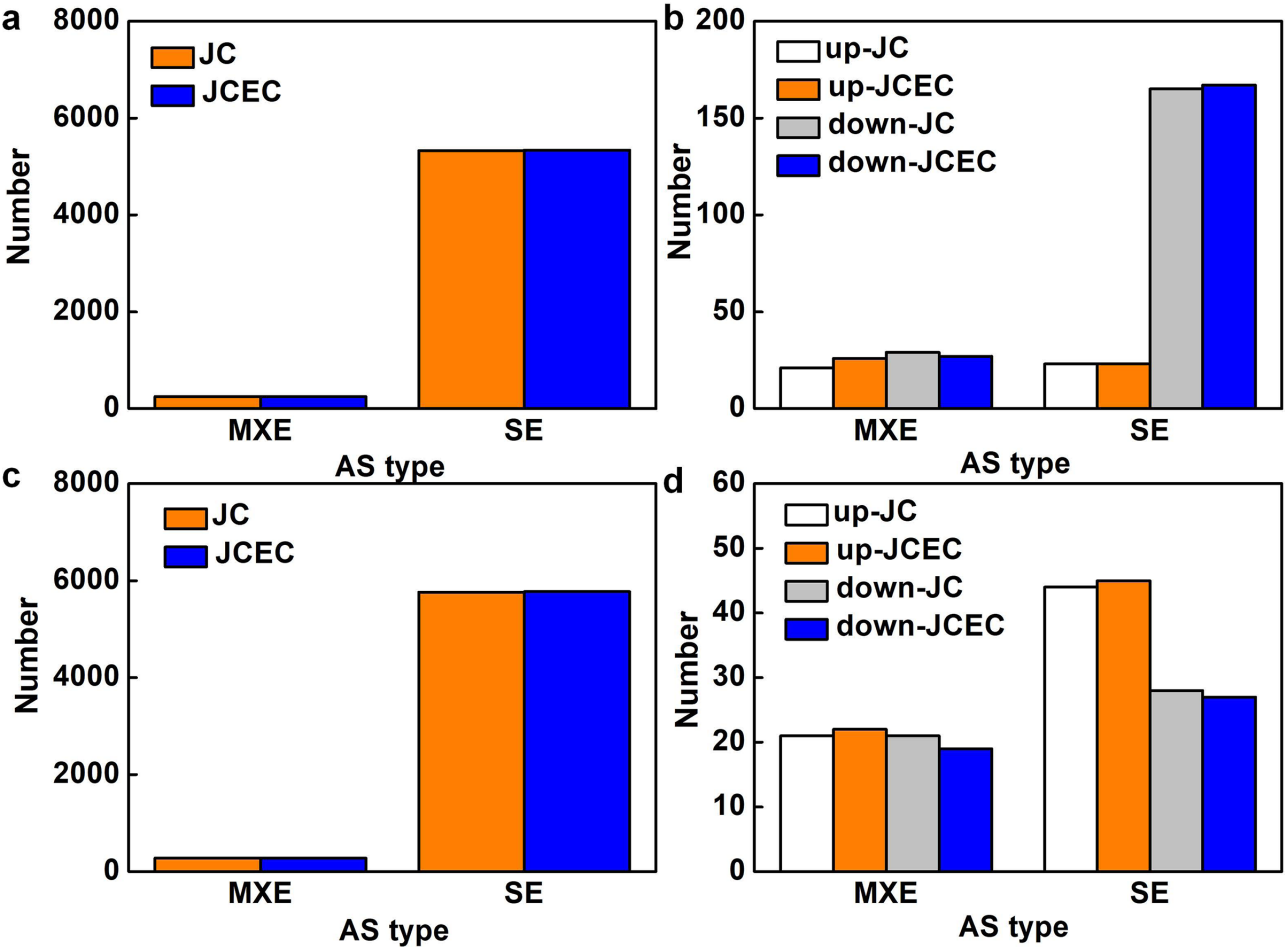
Alternative splicing (AS) events. (A) Total AS events in C_vs_S group. (B) Differentially expressed AS events in in C_vs_S group. (C) Total AS events in S_vs_JS group. Differentially expressed AS events in S_vs_JS group. C_vs_S: salt (S) treatment compared to control (C). S_vs_JS: JA+salt (JS) treatment compared to salt treatment. MXE, mutually exclusive exons; SE, skipped exon; JC, junction counts; JCEC, JC+reads on target.

Following the differential analysis of alternative splicing events, rMATS was used to analyze the categories of alternative splicing events, compute their number, and determine the expression level of each type of alternative splicing event individually for each differential grouping. When detected by junction counts (JC), salt stress resulted in 5,568 alternative splicing (AS) events when compared to control, 95.6% of which were skipped exon (SE). Mutually exclusive exons (MXE) events accounted for 4.4% (Fig. 7A). Among all AS events, 238 events were differentially expressed, with 79.0% SE events (23 upregulated and 165 downregulated) and 21.0% MXE events (21 upregulated and 29 downregulated) (Fig. 7B). Similarly, SE events (95.4%) were predominant when compared JS to S treatment. The rest were MXE events (Fig. 7C). There were 114 differentially expressed AS events caused by JS treatment, with 72 SE and 42 MXE events. Up- and down-regulated MXE events were equal, whereas most SE events were upregulated (Fig. 7D). There was no alternative 5′ splice site (A5SS), alternative 3′ splice site (A3SS) and retained intron (RI) events were detected in our test. Results obtained by JCEC were similar to JC.

We further discovered SE events in various essential genes involved in salt stress response, including transcription factors (bZIPs and AP2), Gluthatione S-transferases, cyclin-dependent kinase, auxin efflux carrier family protein (Table S2). JS treatment resulted in some unique differentially expressed SE events, such as genes related to Heat shock proteins (HSP), ubiquitin-conjugating enzyme, ATP-binding cassette, and transcription factors (MYBs and MYC2) (Table S3).

### Gene expression validation

**Figure 8:**
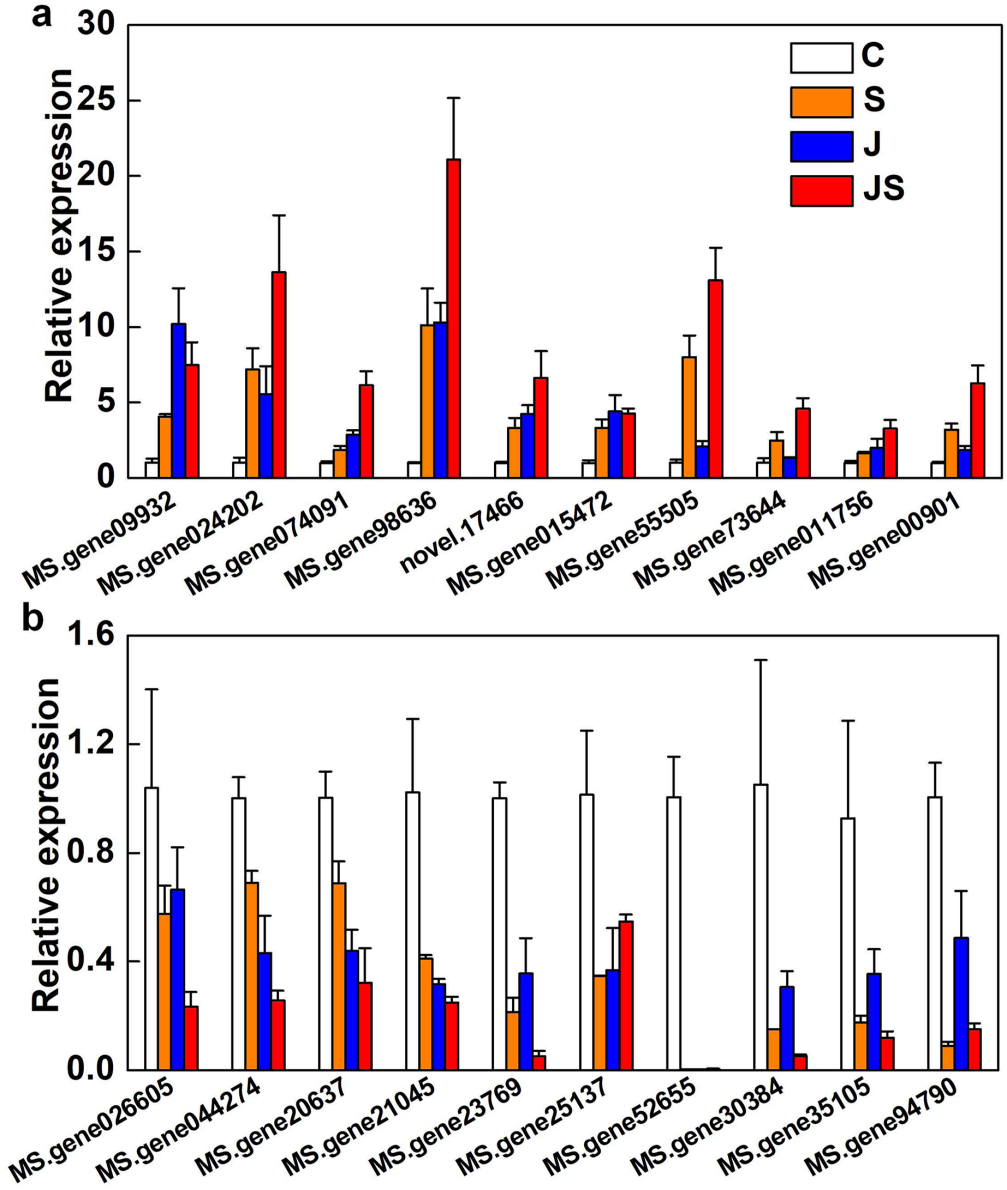
Relative expression of genes co-up-regulated (A) and co-down-regulated (B) by S, J and JS. Data was represented by the means of three replicates ± SE.

qRT-PCR was used to verify the fold change of DEGs. Among the 20 DEGs picked were enzyme genes from the secondary metabolic pathway (MS.gene015472), a new gene (novel.17466), and transcription factors (MS.gene074091, MS.gene94790, MS.gene30384, MS.gene026605, and MS.gene20637). All of the randomly picked genes were identified using PCR. The consistency between the fold change of these DEGs and the RNA-seq results indicates the data’s repeatability (Fig. 8, raw data is shown in Table S4).

## Discussion

Multiple genes control the quantitative trait of salt tolerance in plants. By scanning the expression patterns of these genes, transcriptome analysis may be used to discover the processes and essential genes involved in response to numerous environmental stimuli (Blumenberg, 2019). However, most alfalfa transcriptome studies have only examined expression levels in response to certain factors, such as abiotic or biotic stressors. In this study, transcriptome profiling was performed to establish roles of JA in coping alfalfa salt stress. As a result, we provided a framework on which some novel genes and pathways can be further studied.

The number of DEGs between S *vs* C and JS *vs* S was one of the most notable differences. Most DEGs were upregulated by 150 mM NaCl, which was inconsistent with the previous RNA-seq analysis of 250 mM NaCl treated-alfalfa (Yu et al., 2021). This result indicated that the intensity of salt stress might affect the expression pattern of genes. Intriguingly, most DEGs regulated by JS compared to S were not altered by salt stress alone, meaning that JA combined with salt activated some unique pathways to resist high salinity. A cluster of genes was up- or down-regulated by JA or salt treatment alone, with JS further enhancing this regulation. Accordingly, we inferred that JA primed plants in a resistant state under salt stress.

Salt stress induced changes in plant metabolism, biology and biochemistry, eventually inhibiting plant growth and development (Yang & Guo, 2018). Hydrogen peroxide (H_2_O_2_) is a kind of ROS generated in plant defense responses. Overaccumulation of H_2_O_2_ is the primary cause of oxidative stress (Nadarajah, 2020). We identified that genes related to the response to hydrogen peroxide was one of the most enriched in biological process by salt stress, indicating the production of ROS and activation of antioxidant. However, this catalogue was not enriched in JS *vs* S analysis. Exogenous JA has been proven to significantly decreased the accumulation of H_2_O_2_ in salt-stressed wheat by increasing the activity of antioxidant enzymes (Qiu et al., 2014). Scavenging of H_2_O_2_ during stress responding depends on enzymatic reaction catalyzed by antioxidant enzymes and non-enzymatic reaction composed of antioxidants such as ascorbic acid (AsA) and glutathione (GSH) (Hasanuzzaman et al., 2019). In this study, antioxidant enzymes encoding genes, SOD and POD were inhibited by salt stress, whereas upregulated by JS treatment. Meanwhile, genes engaged in ascorbate and aldarate metabolism were exclusively upregulated by JS treatment compared to S in our KEGG analysis. Therefore, it was inferred that plants under JS treatment might accumulate less H_2_O_2_ than that under salt alone treatment, because of the activation of enzymatic and nonenzymatic reactions to scavenging H_2_O_2_.

Surviving under salinity conditions is an energy-consuming process, along with biosynthesis and degradation of substances. Plants obtain energy though metabolism (Kazem et al., 2020). As expected, the analysis of KEGG pathways indicated that DEGs were primarily enriched in metabolic processes and secondary metabolites biosynthesis, regardless of salt along or combined with JA. Salt-stressed cells tend to change from primary metabolism to secondary metabolism, leading to production of proline, betaine, organic acids, sugar alcohols and other secondary metabolites (Osbourn, 2003). They play varied functions in plant osmotic regulation, enzymes protection and ion transporters biosynthesis (Ahanger et al., 2019; Al Hinai et al., 2022). The common pathways enriched in S *vs* C and JS *vs* S indicated that JS further altered related genes expression, consequently ensuring the energy supply (primary metabolism regulation) or enhancing salt tolerance (secondary metabolism regulation). In addition, we identified the regulation of JS compared to S on the expression of genes related to NADH and NADPH dehydrogenases activity in GO analysis. These enzymes catalyze the oxidase of NADH and NADPH, subsequently generating ATP and providing energy (Fromm et al., 2016).

AS is a post-transcriptional strategy to increase the diversity and complexity of transcripts and proteins (Yang et al., 2022). SE events dominate all AS events in the current test. Some genes significantly enriched in KEGG analysis were identified alternatively spliced. For example, MS.gene037325, an EIN3-binding F-box protein (EFB) which was enriched in MAPK signaling and hormone signal transduction pathway significantly regulated by salt stress rather than JS. *EFB* genes are components of ethylene signal transduction (Liu et al., 2017), indicating that ethylene is involved in alfalfa salt stress response. JS compromised the effects of salt stress on *EFB*, which might be evidenced by the antagonistic roles of JA and ethylene in plant defense response (An et al., 2017). *MYC2*, a master transcription factor of JA signal transduction, was alternatively spliced and upregulated by JS compared to S. Overexpression of *MYC2* increased the sensitivity of JA and induced a series of JA-dependent resisting responses (Kazan & Manners, 2008). The specifically upregulated MYC2 by JA might guarantee the regulation of gene transcription downstream of JA.

## Conclusion

Finally, RNA from four treatments was sequenced to better understand how JA affects alfalfa salt stress response. JA combined with salt treatment further altered a serious genes expression on the basis of salinity alone. These genes concentrated on plant energy metabolism, antioxidant, phytohormone signal transduction and transcription factors. Firstly, S or JA affected genes transcription level involved in maintaining of energy supplying, priming of osmotic and redox regulation. This transcriptional regulation may involve activation of transcription factors. Next, at post-transcriptional level, S or JS induced genes alternative splicing, generating different isoforms. Taken together, this study provided reference data base and potential molecular mechanisms for JA mediated salt tolerance in alfalfa.

## Supplemental Information

10.7717/peerj.15324/supp-1 Supplemental Information 1 Primers used for qRT-PCR.

10.7717/peerj.15324/supp-2 Supplemental Information 2 All differentially expressed genes.

10.7717/peerj.15324/supp-3 Supplemental Information 3 DEGs regulated by S and JS in opposite direction.

10.7717/peerj.15324/supp-4 Supplemental Information 4 DEGs undergo alternative splicing.

10.7717/peerj.15324/supp-5 Supplemental Information 5 Raw data of qRT-PCR in Fig. 8.

10.7717/peerj.15324/supp-6 Supplemental Information 6 Raw data for Fig. 5.

## Additional Information and Declarations

### DNA Deposition

The following information was supplied regarding the deposition of DNA sequences: Sequences are available at NCBI GenBank: PRJNA907206.

### Data Availability

The following information was supplied regarding data availability: The raw data are available in the Supplemental Files.
