# Supplementary material for: Genome-wide transcriptomic analysis identifies candidate genes involved in jasmonic acid-mediated salt tolerance of alfalfa
Source: PeerJ. 2023 May 5;11:e15324. doi: 10.7717/peerj.15324 (PMC10166079; doi:10.7717/peerj.15324)
Supplement: Supplemental Information 1 [file peerj-11-15324-s001.docx]

**Table S1. Gene specific primers used in this study**

| Gene ID | Forward primers | Reverse primers |
| --- | --- | --- |
| MS.gene015472 | ACAATTCCGGCCAACTTCAC | ATTCAGCCCGGTTTCTAAGC |
| MS.gene024202 | CAATTCACCTCCGGTTAGACC | GACGAGCACTATCATCATCCG |
| MS.gene074091 | TCCACTAAAGACCGTCACACC | AAGCCACTCGATCGTTTCAC |
| MS.gene98636 | TGTTGTTCCATTCCCTGATGC | ACTGCTGCTGGTTGTTTCTG |
| novel.17466 | AACCTGCGATCATTGAAGCC | ATTTGCGGCTCTCTTGCTTC |
| MS.gene015472 | AACCTGCGATCATTGAAGCC | ATTTGCGGCTCTCTTGCTTC |
| MS.gene024202 | TCTACTCAACCAAGCGGGAAC | GACGGTGAATTGGACTGAAGG |
| MS.gene074091 | AAGTGCTTTTCGCCGGAAAC | TGCAATCTGCCGTTGTCATG |
| MS.gene98636 | TTATGCCGATGACCATGCAC | TTTGTTGGGCTTGAGCTTGG |
| MS.gene011756 | ATCATCAGCGGCCTTCAAAC | TCGCGGTTTTGATCTTGCTG |
| MS.gene026605 | ATGCCGGTTCAAACGATGAC | AGCTCCCTCGTCAGTTGAAAG |
| MS.gene044274 | ACAAATTGGGCTGTTGCTGAC | TCACCGGTTTCTTTGTGAGC |
| MS.gene20637 | AGCTTCATCACAATGGCCAAG | AGCTGTTGGTACTGAAAGCC |
| Ms.gene21045 | TGACCCAAACCGACAACAAC | TGTGACCTAATTCGCGTGTG |
| MS.gene23769 | ATTTCAAGGTGGACGTGGTG | AACGTTGAAATGGCCAGCTC |
| MS.gene25137 | ATTCGAATGCCAGCGACATG | ATTGAGAAATCGCCGGCTTC |
| MS.gene52655 | ACGTGTCCGAAGAGTCACAC | TTGCAGGTTCAGCTTGTTCC |
| MS.gene30384 | ACAATTCCGGCCAACTTCAC | ATTCAGCCCGGTTTCTAAGC |
| MS.gene35105 | CGCTGGACCACAAATGACAG | TGTTGCTGACAGTGTGACTC |
| MS.gene94790 | AAACAATTCGCTGGCTCCTC | TTTGAGTGTGCCGTTGACTG |
| MsACTIN2 | TCAATGTGCCTGCCATGTATGT | ACTCACACCGTCACCAGAATCC |
